# Characterization of novel nonacid glycosphingolipids as biomarkers of human gastric adenocarcinoma

**DOI:** 10.1016/j.jbc.2022.101732

**Published:** 2022-02-15

**Authors:** Chunsheng Jin, Susann Teneberg

**Affiliations:** Department of Medical Biochemistry and Cell Biology, Institute of Biomedicine, Sahlgrenska Academy, University of Gothenburg, Göteborg, Sweden

**Keywords:** gastric adenocarcinoma, glycosphingolipid characterization, mass spectrometry, P1 glycosphingolipid, *H. pylori* binding, BabA, blood group antigen–binding adhesion, CagA, cytotoxin-associated antigen A, LabA, LacdiNAc–binding adhesion, LC, liquid chromatography, LTQ, linear trap quadropole, MS, mass spectrometry, SabA, sialic acid–binding adhesion, VacA, vacuolating cytotoxin A

## Abstract

Changes in glycosphingolipid structures have been shown to occur during the development of several types of human cancers, generating cancer-specific carbohydrate structures that could be used as biomarkers for diagnosis and therapeutic targeting. In this study, we characterized nonacid glycosphingolipids isolated from a human gastric adenocarcinoma by mass spectrometry, enzymatic hydrolysis, and by binding with a battery of carbohydrate-recognizing ligands. We show that the majority of the complex nonacid glycosphingolipids had type 2 (Galβ4GlcNAc) core chains (neolactotetraosylceramide, the Le^x^, H type 2, x_2_, and the P1 pentaosylceramides, and the Le^y^, A type 2, and neolacto hexaosylceramides). We also found glycosphingolipids with type 1 (Galβ3GlcNAc) core (lactotetraosylceramide and the H type 1 pentaosylceramide) and globo (GalαGal) core chains (globotriaosylceramide and globotetraosylceramide). Interestingly, we characterized two complex glycosphingolipids as a P1 heptaosylceramide (Galα4Galβ4GlcNAcβ3Galβ4GlcNAcβ3Gal β4Glcβ1Cer) and a branched P1 decaosylceramide (Galα4Gal β4GlcNAcβ3(Galα4Galβ4GlcNAcβ6)Galβ4GlcNAcβ3Galβ4Glc β1Cer). These are novel glycosphingolipid structures and the first reported cases of complex glycosphingolipids larger than pentaosylceramide carrying the P1 trisaccharide. We propose that these P1 glycosphingolipids may represent potential biomarkers for the early diagnosis of gastric cancer.

Gastric adenocarcinoma remains a common cause of cancer death worldwide. In 2020, there were 1.09 million new cases and 769,000 deaths because of stomach cancer (https://www.who.int/news-room/fact-sheets/detail/cancer). Today, almost two-thirds of the stomach cancer cases are found in developing countries. The disease is often diagnosed at a late stage, and the 5-year survival rate is low, in most countries, not more than 15%. It is now well established that chronic *Helicobacter pylori* infection predisposes individuals toward gastric adenocarcinoma later in life (reviewed in Ref. (1)), and the International Agency of Research on Cancer at the World Health Organization at an early stage classified *H. pylori* as a class I carcinogen. *H. pylori* infection leads to inflammatory changes in the gastric epithelium, and initially causes an acute gastritis, which is followed by chronic gastritis. Subsequently, more degenerative changes appear leading to premalignant conditions as atrofic gastritis, metaplasia, and dysplasia.

*H. pylori* colonization of the human stomach is initiated by binding of bacterial adhesins to carbohydrate receptors on the gastric epithelium. A number of different carbohydrate receptor candidates (*e.g.*, gangliotetraosylceramide, the Le^b^ blood group determinant, sulfatide, lactosylceramide, neolacto sequences, lactotetraosylceramide, sialyl-Le^x^, and related sequences) have been reported (reviewed in Ref. (2)). Despite the multitude of candidate *H. pylori* glycan receptors, only three carbohydrate-binding adhesins have been characterized to date; the blood group antigen–binding BabA adhesin, the sialic acid–binding SabA adhesin, and the LabA adhesin (reviewed in Ref. (3)).

The first *H. pylori* adhesin identified was the Le^b^-binding adhesin BabA (4). *H. pylori* strains expressing BabA together with VacA and CagA (triple-positive strains) are highly associated with severe gastric diseases, as peptic ulcer or gastric adenocarcinoma. BabA mediates the initial attachment of *H. pylori* to the human gastric mucosa. The first observation that the fucosylated blood group antigens H type 1 and Le^b^ are recognized by *H. pylori* BabA was followed by a division of BabA-producing *H. pylori* strains into specialist and generalist strains, depending on their mode of binding to Le^b^ and related carbohydrate sequences (5). BabA of specialist strains binds only to glycoconjugates with unsubstituted terminal Fucα2Gal sequence as the H type 1 and Le^b^ determinants, whereas the generalist BabA tolerates an addition of αGal or αGalNAc to the Gal, as in the blood group A or B type 1 determinants. Thereafter, it was demonstrated that BabA binds to blood group O and A determinants on type 4 core chains (Globo H and Globo A), in addition to blood group determinants on type 1 core chains (6). The structural basis of the different binding modes of BabA was recently determined by X-ray crystallography of the adhesin domain of specialist and generalist BabA, alone and in complex with ABO/Le^b^ oligosaccharides (7).

SabA, the sialic acid–binding adhesin of *H. pylori*, binds to sialylated glycoconjugates, such as sialyl-Le^x^ and sialyl-Le^a^ (8). The inflammatory response that follows *H. pylori* colonization of the human gastric mucosa leads to increased expression of sialylated glycans, that is, an increased density of attachment points for the bacteria.

The LabA adhesin was initially reported to bind to LacDiNAc sequences on mucins (9). However, more recent studies have failed to confirm this interaction (10, 11). Thus, further studies are required to elucidate the carbohydrate-binding specificity of LabA.

We have recently characterized the acid and nonacid glycosphingolipids of the normal human stomach (12, 13). Acid glycosphingolipids recognized by *H. pylori* SabA were Neu5Acα3-neolactohexaosylceramide and Neu5Acα3-neolactooctaosylceramide (12), and the presence of these two SabA ligands in human gastric adenocarcinoma has previously been reported (14). Among the nonacid glycosphingolipids, there were several ligands for BabA-mediated binding of *H. pylori* (Le^b^ hexaosylceramide, H type 1 pentaosylceramide, and A type 1/ALe^b^ heptaosylceramide; summarized in Table 1) (13). Other *H. pylori*-binding glycosphingolipids, recognized by BabA-deficient strains, were lactosylceramide, lactotetraosylceramide, the x_2_ pentaosylceramide, and neolactohexaosylceramide (Table 1).

**Table 1:** *H. pylori* binding nonacid glycosphingolipids in healthy human stomachs^a^

Trivial name	Glycosphingolipid structure	BabA binding^b^
Lactosylceramide^c^	Galβ4Glcβ1Cer^d^	−
Lactotetra	Galβ3GlcNAcβ3Galβ4Glcβ1Cer	−
Neolactotetra	Galβ4GlcNAcβ3Galβ4Glcβ1Cer	−
H type 1 penta	Fucα2Galβ3GlcNAcβ3Galβ4Glcβ1Cer	+
x_2_ penta	GalNAcβ3Galβ4GlcNAcβ3Galβ4Glcβ1Cer	−
Le^b^ hexa	Fucα2Galβ3(Fucα4)GlcNAcβ3Galβ4Glcβ1Cer	+
Neolactohexa	Galβ4GlcNAcβ3Galβ4GlcNAcβ3Galβ4Glcβ1Cer	−
A type 1/ALe^b^ hepta	GalNAcα3(Fucα2)Galβ3(Fucα4)GlcNAcβ3Galβ4Glcβ1Cer	+

a Data from Ref. (13).

b Recognized by generalist BabA.

c Binding to lactosylceramide with phytosphingosine and/or hydroxy fatty acids.

d Only the glycan part is shown in the symbolic structures, which are depicted using the Symbol Nomenclature for Glycomics (56, 57).

In the present study, nonacid glycosphingolipids isolated from one human gastric adenocarcinoma specimen were characterized by mass spectrometry (MS), enzymatic hydrolysis, and by binding of a battery of carbohydrate-recognizing ligands, with special attention to compounds recognized by *H. pylori.*

## Results

### Isolation of human gastric adenocarcinoma glycosphingolipids

**Figure 1:**
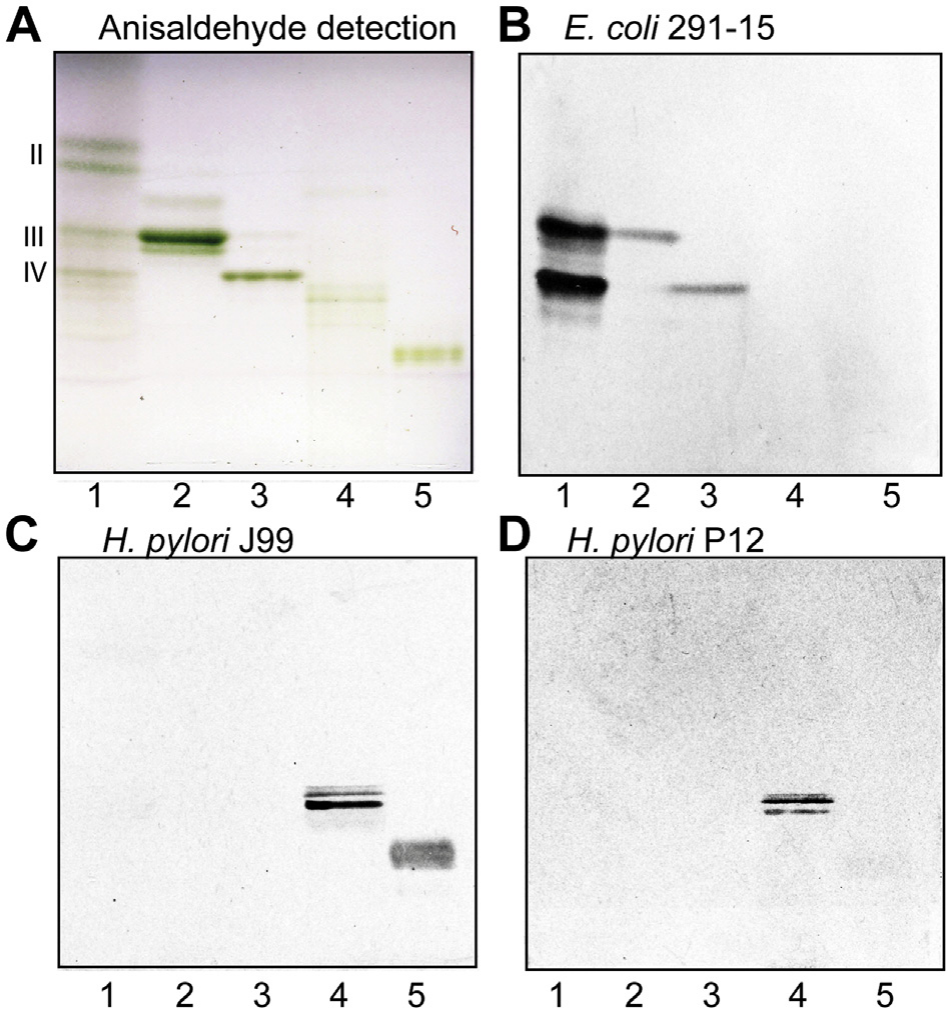
**Binding of *Helicobacter pylori* and P-fimbriated *Escherichia coli* to the total nonacid glycosphingolipids of human gastric adenocarcinoma.** Thin-layer chromatogram detected with anisaldehyde (*A*), and autoradiograms obtained by binding of recombinant *E. coli* strain 291-15 (*B*), *H. pylori* strain J99 (*C*), and *H. pylori* strain P12 (*D*). The glycosphingolipids were separated on aluminum-backed silica gel plates, using chloroform/methanol/water 60:35:8 (by volume) as solvent system, and the binding assays were performed as described under the “Experimental procedures” section. The lanes were: lane 1, nonacid glycosphingolipids of human gastric adenocarcinoma, 80 μg; lane 2, reference globotriaosylceramide (Galα4Galβ4Glcβ1Cer), 4 μg; lane 3, reference globotetraosylceramide (GalNAcβ3Galα4Galβ4Glcβ1Cer), 4 μg; lane 4, reference lactotetraosylceramide (Galβ3GlcNAcβ3Galβ4Glcβ1Cer), 4 μg; and lane 5, reference Le^b^ hexaosylceramide (Fucα2Galβ3(Fucα4)GlcNAcβ3Galβ4Glcβ1Cer), 4 μg. The *Roman numbers* to the *left* of the chromatogram in (*A*) denote the approximate number of carbohydrate units in the bands.

Total acid and nonacid glycosphingolipid fractions were isolated from a human gastric adenocarcinoma as described previously (14). Thereby, 430 mg of total neutral glycosphingolipids were obtained from 80 g of starting material. The major part of the nonacid fraction was used for other studies, leaving 30 mg for the structural characterization reported here. Thin-layer chromatography with anisaldehyde staining demonstrated the presence of three major glycosphingolipids in the total nonacid fraction (Fig. 1*A*, lane 1). These compounds migrated as diaosylceramides, triaosylceramides, and tetraosylceramides, respectively. A number of low-abundant compounds migrating below the tetraglycosylceramide region were also present.

### Characterization of the total nonacid glycosphingolipid fraction from human gastric adenocarcinoma

#### Binding of *H. pylori* and P-fimbriated *Escherichia coli*

First, we examined the binding of BabA expressing/Le^b^ binding *H. pylori* strain J99, lactotetraosylceramide binding *H. pylori* strain P12, and Galα4Gal binding/P-fimbriated *Escherichia coli* strain 291-15 to the total nonacid glycosphingolipid fraction from human gastric adenocarcinoma. Here, a distinct binding of the Galα4Gal recognizing *E. coli* was obtained (Fig. 1*B*, lane 1). The compounds recognized by the bacteria comigrated with reference globotriaosylceramide and globotetraosylceramide (Fig. 1*B*, lanes 2 and 3). There was also a weak binding to a compound migrating below globotetraosylceramide (Fig. 1*B*, lane 1).

There was no binding of the Le^b^ recognizing *H. pylori* strain J99 (Fig. 1*C*, lane 1) or the lactotetraosylceramide binding *H. pylori* strain P12 (Fig. 1*D*, lane 1) to the total nonacid glycosphingolipid fraction from human gastric adenocarcinoma, although both bacteria properly recognized reference lactotetraosylceramide (Fig. 1, *C* and *D*, lane 4), and in the case of the J99 strain, binding to reference Le^b^ hexaosylceramide was also obtained (Fig. 1*C*, lane 5).

#### Liquid chromatography–electrospray ionization/MS of glycosphingolipid-derived oligosaccharides

Thereafter, the glycosphingolipids in the total nonacid glycosphingolipid fraction were characterized by MS. The total nonacid fraction was hydrolyzed with endoglycoceramidase II from *Rhodococcus* sp., and the oligosaccharides thereby obtained were characterized by LC–ESI/MS using a graphitized carbon column. This gives a resolution of isomeric oligosaccharides, and by MS^2^, a series of C-type ions is obtained, which gives the carbohydrate sequence (15). Furthermore, the MS^2^ spectra of oligosaccharides with a Hex or HexNAc substituted at C-4 have diagnostic crossring ^0,2^A-type and ^2,4^A-type fragment ions, which allow identification of linkage positions (15, 16). Thus, such fragment ions are present in the MS^2^ spectra of oligosaccharides with globo (Galα4Gal) or type 2 (Galβ4GlcNAc) core structures but not in the MS^2^ spectra obtained from oligosaccharides with isoglobo (Galα3Gal) or type 1 (Galβ3GlcNAc) core chains. Comparison of retention times and MS^2^ spectra of oligosaccharides from reference glycosphingolipids is also used for identification of oligosaccharides.

**Figure 2:**
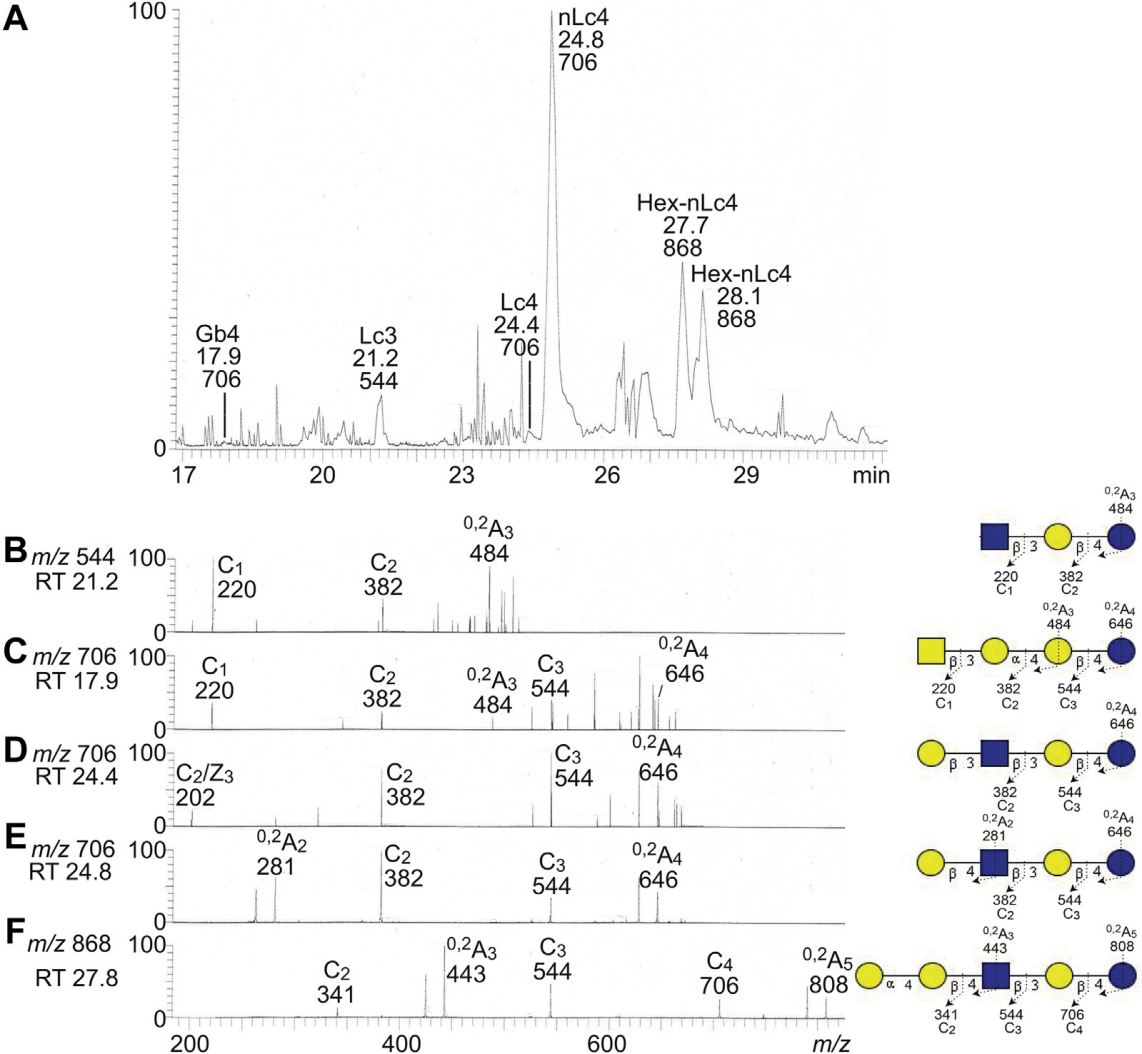
**LC–ESI/MS of the oligosaccharides derived from the total nonacid glycosphingolipid fraction from human gastric adenocarcinoma by hydrolysis with endoglycoceramidase II from *Rhodococcus* spp.** The identification of oligosaccharides was based on their retention times, determined molecular masses, and subsequent MS^2^ sequencing. *A*, base peak chromatogram from LC–ESI/MS of the oligosaccharides obtained from the total nonacid glycosphingolipid fraction from human gastric cancer. *B*, MS^2^ of the ion at *m/z* 544 at retention time 20.4 min. *C*, MS^2^ of the ion at *m/z* 706 at retention time 17.9 min. *D*, MS^2^ of the ion at *m/z* 706 at retention time 24.4 min. *E*, MS^2^ of the ion at *m/z* 706 at retention time 24.8 min. *F*, MS^2^ of the ion at *m/z* 868 at retention time 27.8 min. The proposed structures in the interpretation formulas are depicted at the right side using the Symbol Nomenclature for Glycomics (SNFG) (56, 57), and nomenclature of fragments was defined by Domon and Costello (58). The oligosaccharides identified in the chromatogram were: Gb4, GalNAcβ3Galα4Galβ4Glc; Lc3, GlcNAcβ3Galβ4Glc; Lc4, Galβ3GlcNAcβ3Galβ4Glc; nLc4, Galβ4GlcNAcβ3Galβ4Glc; Hex-nLc4; Hex-Galβ4GlcNAcβ3Galβ4Glc. *Y*-axis, relative intensity. ESI, electrospray ionization; LC, liquid chromatography; MS, mass spectrometry; RT, retention time.

The base peak chromatogram from LC–ESI/MS of the oligosaccharides obtained from the total nonacid glycosphingolipid fraction from the human gastric adenocarcinoma had five molecular ions corresponding to oligosaccharides ranging from trisaccharides (detected as [M–H^+^]^−^ ions at *m/z* 544) to pentasaccharides (detected as [M–H^+^]^−^ ions at *m/z* 868) (Fig. 2*A*).

MS^2^ of the molecular ion at *m/z* 544 gave prominent C-type fragment ions (C_1_ at *m/z* 220 and C_2_ at *m/z* 382) identifying a HexNAc-Hex-Hex sequence (Fig. 2*B*). There was no ^0,2^A_2_ fragment ion at *m/z* 322, as in the ganglio trisaccharide. Thus, a lacto trisaccharide (GlcNAcβ3Galβ4Glc) was tentatively identified.

The base peak chromatogram had three molecular ions at *m/z* 706, eluting at 17.9, 24.4, and 24.8 min, respectively. The MS^2^ spectrum of the minor molecular ion at *m/z* 706 at retention time 17.9 min (Fig. 2*C*) had a C-type fragment ion series (C_1_ at *m/z* 220, C_2_ at *m/z* 382, and C_3_ at *m/z* 544), demonstrating a HexNAc-Hex-Hex-Hex sequence. The ^0,2^A_3_ fragment ion at *m/z* 484 demonstrated a 4-substituted Hex (15, 16). Taken together, this allowed identification of a globo tetrasaccharide (GalNAcβ3Galα4Galβ4Glc).

MS^2^ of the ion at *m/z* 706 at the retention time 19.6 min allowed identification of a lacto tetrasaccharide (Galβ3GlcNAcβ3Galβ4Glc) (Fig. 2*D*). This was concluded from the C-type fragment ions (C_2_ at *m/z* 382 and C_3_ at *m/z* 544) identifying a Hex-HexNAc-Hex-Hex sequence, along with the C_2_/Z_3_ ion (D_1–2_ ion) at *m/z* 202, obtained by a C_2_–Z_3_ double cleavage, and diagnostic for a 3-substituted HexNAc, that is a type 1 chain (16).

The molecular ion at *m/z* 706 at the retention time 24.8 min was the major ion in the base chromatogram. MS^2^ of this ion also gave a series of C-type fragment ions (C_2_ at *m/z* 382 and C_3_ at *m/z* 544) identifying a Hex-HexNAc-Hex-Hex sequence (Fig. 2*E*). In addition, this spectrum had a ^0,2^A_2_ fragment ion at *m/z* 281 demonstrating a terminal Hex-HexNAc sequence with a 4-substituted HexNAc, that is, a type 2 chain (15, 16). Thus, a neolacto tetrasaccharide (Galβ4GlcNAcβ3Galβ4Glc) was characterized.

Finally, a Hex-Hex-HexNAc-Hex-Hex sequence was identified by the series of C-type fragment ions (C_2_ at *m/z* 341, C_3_ at *m/z* 544, and C_4_ at *m/z* 706) obtained by MS^2^ of the ion at *m/z* 868 eluting at 27.8 to 28.1 min (Fig. 2*F*). Here, 4-substitution of the internal HexNAc was demonstrated by the ^0,2^A_3_ fragment ion at *m/z* 443 (15, 16). Taken together, this demonstrated neolacto tetrasaccharide substituted with a terminal Hex (Hex-Galβ4GlcNAcβ3Galβ4Glc).

**Figure 3:**
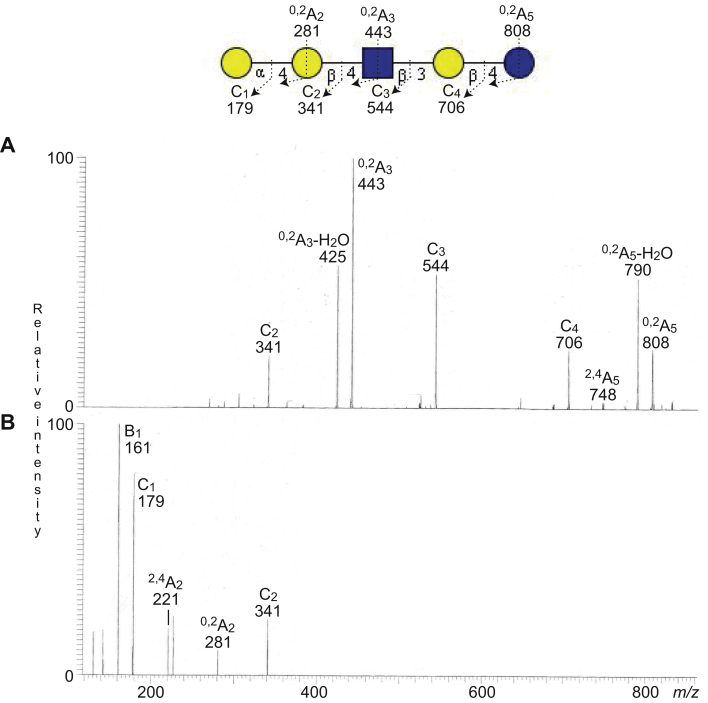
**LC–ESI/MS of the oligosaccharides derived from the total nonacid glycosphingolipid fraction from human gastric adenocarcinoma by hydrolysis with endoglycoceramidase II from *Rhodococcus* spp.** Reanalysis with MS^3^. The identification of oligosaccharides was based on their retention times, determined molecular masses, and subsequent MS^2^ sequencing. *A*, MS^2^ of the ion at *m/z* 868. *B*, MS^3^ of the ion at *m/z* 443 in (*A*). The proposed structure in the interpretation formula is depicted using the Symbol Nomenclature for Glycomics (SNFG) (56, 57), and nomenclature of fragments was defined by Domon and Costello (58). ESI, electrospray ionization; LC, liquid chromatography; MS, mass spectrometry.

To further characterize the Hex-nLc4 pentasaccharide ion at *m/z* 868, the oligosaccharide sample was analyzed by liquid chromatography (LC)–ESI/MS once again and subjected to MS^3^ (Fig. 3). MS^3^ of the ion at *m/z* 443 gave a distinct ^0,2^A_2_ fragment ion at *m/z* 281 and a ^2,4^A_2_ fragment ion at *m/z* 221, demonstrating that the subterminal Hex was substituted at C-4 (15, 16). Thereby, a P1 pentasaccharide (Galα4Galβ4GlcNAcβ3Galβ4Glc) was tentatively identified.

In summary, LC–ESI/MS of the oligosaccharides derived from the total nonacid glycosphingolipid fraction from human gastric adenocarcinoma gave identification of a lacto trisaccharide, globo tetrasaccharide, lacto tetrasaccharide, and neolacto tetrasaccharide, and a P1 pentasaccharide.

### Separation of the total nonacid glycosphingolipids from human gastric adenocarcinoma

**Figure 4:**
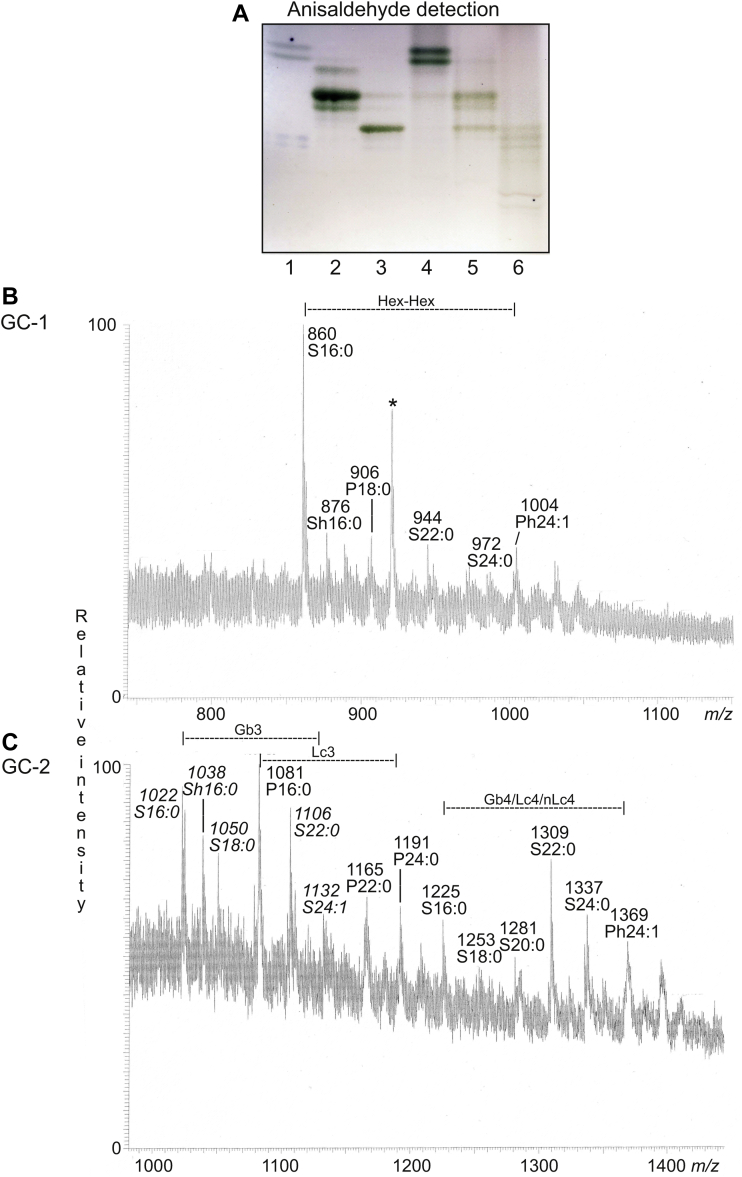
**Characterization of fractions GC-1 and GC-2.***A*, thin-layer chromatogram detected with anisaldehyde. The glycosphingolipids were separated on glass-backed silica gel plates, using chloroform/methanol/water 60:35:8 (by volume) as solvent system. The lanes were: lane 1, reference lactosylceramide (Galβ4Glcβ1Cer), 4 μg; lane 2, reference globotriaosylceramide (Galα4Galβ4Glcβ1Cer), 4 μg; lane 3, reference globotetraosylceramide (GalNAcβ3Galα4Galβ4Glcβ1Cer), 4 μg; lane 4, fraction GC-1, 4 μg; lane 5, fraction GC-2, 4 μg; and lane 6, fraction GC-3, 4 μg. *B*, molecular ion profile from LC–ESI/MS of fraction GC-1. *C*, molecular ion profile from LC–ESI/MS of fraction GC-2. Ions from trihexosylceramide are in italics. The peak marked with a ∗ symbol is a nonglycosphingolipid contaminant. In the shorthand nomenclature for fatty acids and bases, the number before the colon refers to the carbon chain length and the number after the colon gives the total number of double bonds in the molecule. Fatty acids with a 2-hydroxy group are denoted by the prefix h before the abbreviation, as, for example, h16:0. S designates sphingosine (d18:1) long-chain base, and P designates phytosphingosine (t18:0) long-chain base. Gb3, Galα4Galβ4Glcβ1Cer; Lc3, GlcNAcβ3Galβ4Glcβ1Cer; Gb4, GalNAcβ3Galα4Galβ4Glcβ1Cer; Lc4, Galβ3GlcNAcβ3Galβ4Glcβ1Cer; nLc4, Galβ4GlcNAcβ3Galβ4Glcβ1Cer. ESI, electrospray ionization; LC, liquid chromatography; MS, mass spectrometry.

To enrich the slow-migrating glycosphingolipids, the total nonacid glycosphingolipid fraction was next separated on an Iatrobeads column (Iatron Labs). Thereby, three glycosphingolipid-containing fractions were obtained. These fractions were denoted fractions GC-1, GC-2, and GC-3, respectively. The glycosphingolipids in fraction GC-1 migrated in the dihexosylceramide region, whereas fraction GC-2 had glycosphingolipids migrating as triaosylceramides and tetraosylceramides, and fraction GC-3 contained tetraosylceramides and larger compounds (Fig. 4, lanes 4–6).

### LC–ESI/MS of fractions GC-1 and GC-2

The native fractions GC-1 and GC-2 were analyzed by LC–ESI/MS using a polyamine column. Thereby, dihexosylceramides with both sphingosine and phytosphingosine, and both hydroxy and nonhydroxy fatty acids with 16 to 24 carbon atoms, were identified in fraction GC-1 (Fig. 4*B*). Fraction GC-2 had triaosylceramides and tetraosylceramides, also with a mixed population of ceramide species with both sphingosine and phytosphingosine, and both hydroxy and nonhydroxy fatty acids with 16 to 24 carbon atoms (Fig. 4*C*).

### LC–ESI/MS of fraction GC-3

For characterization of fraction GC-3, an aliquot of this fraction was hydrolyzed with endoglycoceramidase II from *Rhodococcus* sp., followed by LC–ESI/MS of the oligosaccharides using a graphitized carbon column. The base peak chromatogram thereby obtained (Fig. S1*A*) had two predominant molecular ions at *m/z* 706 and *m/z* 868, and MS^2^ of these gave identification of a neolacto tetrasaccharide and a P1 pentasaccharide, as aforementioned (data not shown). There were also a number of minor molecular ions, which were found by reconstructed ion chromatograms (Fig. S1, *B*–*G*). Thus, there were three molecular ions at *m/z* 852, eluting at 18.2 min, 21.5 and 24.6 min, and also a number of minor molecular ions at *m/z* 909, *m/z* 998, *m/z* 1055, *m/z* 1071, and *m/z* 1233. There were also a minor doubly charged molecular ion at *m/z* 880 (corresponding to a singly charged ion at *m/z* 1760).

**Figure 5:**
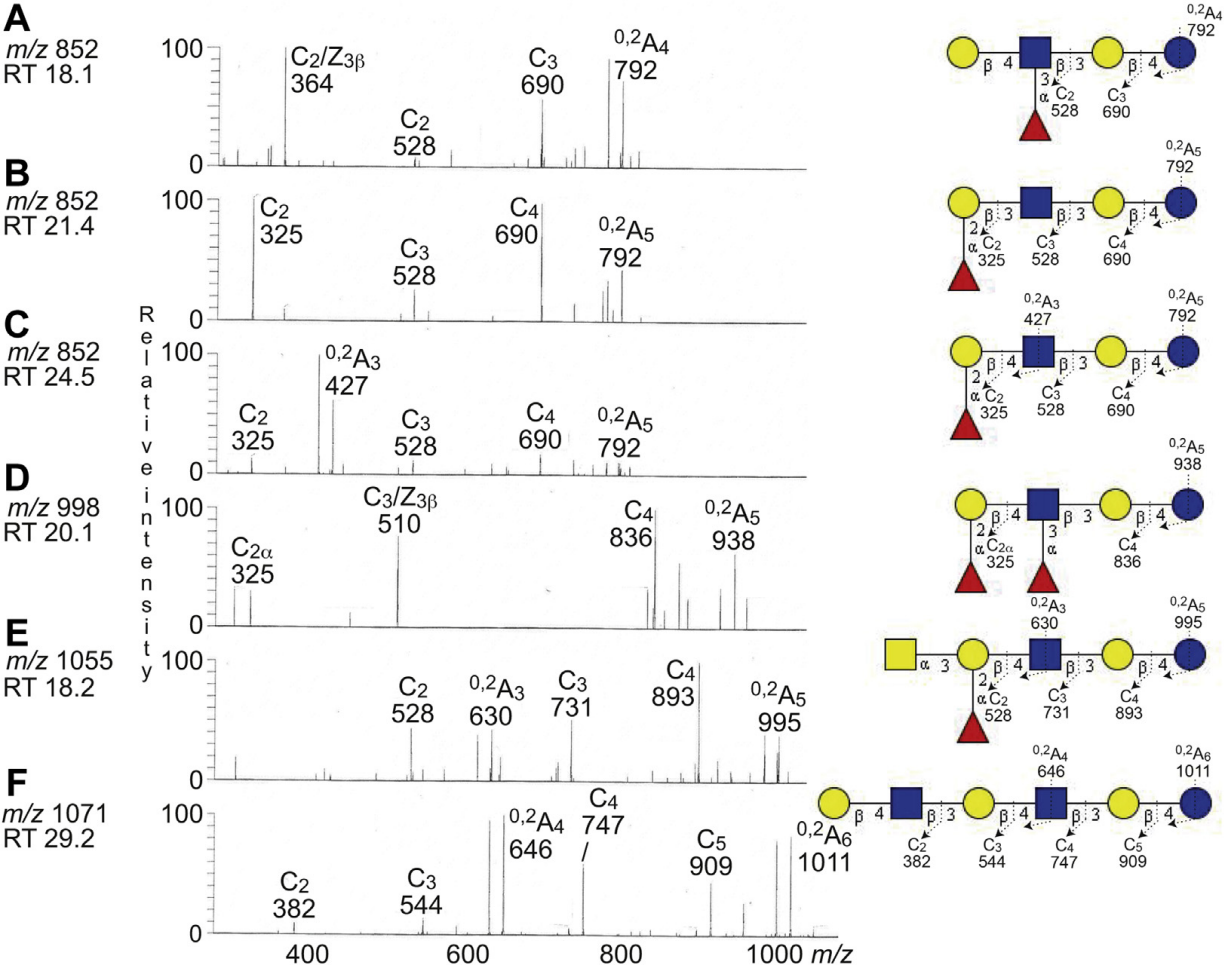
**LC–ESI/MS of the oligosaccharides derived from fraction GC-3 by hydrolysis with endoglycoceramidase II from *Rhodococcus* spp.** The identification of oligosaccharides was based on their retention times, determined molecular masses, and subsequent MS^2^ sequencing. *A*, MS^2^ of the ion at *m/z* 852 at retention time 18.2 min. *B*, MS^2^ of the ion at *m/z* 852 at retention time 21.5 min. *C*, MS^2^ of the ion at *m/z* 852 at retention time 24.6 min. *D*, MS^2^ of the ion at *m/z* 998 at retention time 20.1 min. *E*, MS^2^ of the ion at *m/z* 1055 at retention time 18.1 min. *F*, MS^2^ of the ion at *m/z* 1071 at retention time 29.2 min. See Fig. S1 for base peak chromatogram. The proposed structures in the interpretation formulas are depicted at the *right side* using the Symbol Nomenclature for Glycomics (SNFG) (56, 57), and nomenclature of fragments was defined by Domon and Costello (58). ESI, electrospray ionization; LC, liquid chromatography; MS, mass spectrometry; RT, retention time.

A molecular ion at *m/z* 852 is consistent with a pentasaccharide with one Fuc, one HexNAc, and three Hex. MS^2^ of the ion at *m/z* 852 eluting at 18.2 min gave a spectrum with a dominant ion at *m/z* 364 (Fig. 5*A*). This fragment ion is diagnostic for an internal 4-linked GlcNAc substituted with a Fuc at 3-position and is due to a double glycosidic cleavage of the 3-linked branch (C_2_/Z_3β_) (16). There was also a C_2_ ion at *m/z* 528, and a C_3_ ion at *m/z* 690, and together these spectral features identified a Le^x^ pentasaccharide (Galβ4(Fucα3)GlcNAcβ3Galβ4Glc).

The MS^2^ spectrum of the ion at *m/z* 852 eluting at 21.5 min was distinctly different (Fig. 5*B*) and had a series of C-type fragment ions (C_2_ at *m/z* 325, C_3_ at *m/z* 528, and C_4_ at *m/z* 690), identifying a pentasaccharide with Fuc-Hex-HexNAc-Hex-Hex sequence. This demonstrated an H type 1 pentasaccharide (Fucα2Galβ3GlcNAcβ3Galβ4Glc).

The same series of C-type fragment ions (C_2_ at *m/z* 325, C_3_ at *m/z* 528, and C_4_ at *m/z* 690), identifying a pentasaccharide with Fuc-Hex-HexNAc-Hex-Hex sequence, were present in the spectrum obtained by MS^2^ of the ion at *m/z* 852 eluting at 24.6 min (Fig. 5*C*). This spectrum also had a ^0,2^A_3_ fragment ion at *m/z* 427, which is characteristic for 4-substituted HexNAc, that is, a type 2 carbohydrate chain (15, 16). Thus, an H type 2 pentasaccharide (Fucα2Galβ4GlcNAcβ3Galβ4Glc) was identified.

MS^2^ of the ion at *m/z* 998 demonstrated a Le^y^ hexasaccharide (Fucα2Galβ4(Fucα3)GlcNAcβ3Galβ4Glc) (Fig. 5*D*). This conclusion was based on the prominent ion at *m/z* 510, which is obtained by double glycosidic cleavage of the 3-linked branch at C_3_ and Z_3β_, and is a signature ion for an internal 4-linked GlcNAc substituted with a Fuc at 3-position (16), together with the C-type fragment ions (C_2α_ at *m/z* 325 and C_4_ at *m/z* 836).

MS^2^ of the molecular ion at *m/z* 1055 (Fig. 5*E*) gave a series of C-type fragment ions (C_2_ at *m/z* 528, C_3_ at *m/z* 731, and C_4_ at *m/z* 893) demonstrating a HexNAc-(Fuc-)Hex-HexNAc-Hex-Hex sequence. A type 2 core chain was identified by the ^0,2^A_4_ ion at *m/z* 630. Taken together, this identified a blood group A type 2 hexasaccharide (GalNAcα3(Fucα2)Galβ4GlcNAcβ3Galβ4Glc).

A neolacto hexasaccharide (Galβ4GlcNAcβ3Galβ4GlcNAcβ3Galβ4Glc) was characterized by MS^2^ of the ion at *m/z* 1071 (Fig. 5*F*). This was deduced from the C-type fragment ion series (C_2_ at *m/z* 382, C_3_ at *m/z* 544, C_4_ at *m/z* 747, and C_5_ at *m/z* 909), demonstrating a Hex-HexNAc-Hex-HexNAc-Hex-Hex carbohydrate sequence, along with the ^0,2^A_4_ fragment ion at *m/z* 646, which demonstrated 4-substitution of the innermost HexNAc.

**Figure 6:**
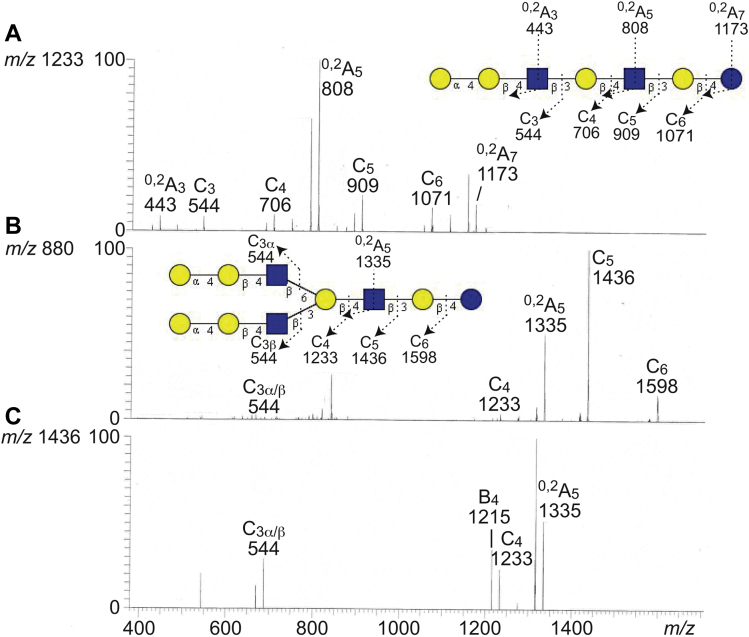
**LC–ESI/MS of the oligosaccharides derived from fraction GC-3 by hydrolysis with endoglycoceramidase II from *Rhodococcus* spp.** The identification of oligosaccharides was based on their retention times, determined molecular masses, and subsequent MS^2^ sequencing. *A*, MS^2^ of the ion at *m/z* 1233 at retention time 31.1 min. *B*, MS^2^ of the ion at *m/z* 880 at retention time 32.8 min. *C*, MS^3^ of the ion at *m/z* 1436 in (*B*). The proposed structures in the interpretation formulas are depicted using the Symbol Nomenclature for Glycomics (SNFG) (56, 57), and nomenclature of fragments was defined by Domon and Costello (58). ESI, electrospray ionization; LC, liquid chromatography; MS, mass spectrometry.

A molecular ion at *m/z* 1233 corresponds to a heptasaccharide with two HexNAc and five Hex. The MS^2^ spectrum obtained of *m/z* 1233 was relatively weak (Fig. 6*A*) and had a series of the C-type fragment ions (C_3_ at *m/z* 544, C_4_ at *m/z* 706, C_5_ at *m/z* 909, and C_6_ at *m/z* 1071) in line with a Hex-Hex-HexNAc-Hex-HexNAc-Hex-Hex heptasaccharide. The ^0,2^A_5_ ion at *m/z* 808 demonstrated 4-substitution of the innermost HexNAc. Taken together, these spectral features gave a tentative identification of a P1 heptasaccharide (Galα4Galβ4GlcNAcβ3Galβ4GlcNAcβ3Galβ4Glc).

A molecular ion at *m/z* 1760 is consistent with decasaccharide composed of three HexNAc and seven Hex. The spectrum from MS^2^ of the doubly charged molecular ion at *m/z* 880 (corresponding to a singly charged ion at *m/z* 1760) (Fig. 6*B*) was a typical MS^2^ spectrum of a branched oligosaccharide, with predominant C-type ions from the reducing end and relatively weak ions from the nonreducing end (6). The C_3α/β_ ion at *m/z* 544 indicated Hex-Hex-HexNAc terminals (Fig. 6, *B* and *C*), and taken together with the C-type ions at *m/z* 1233 (C_4_), *m/z* 1436 (C_5_), and *m/z* 1598 (C_6_), this indicated a Hex-Hex-HexNAc-(Hex-Hex-HexNAc)Hex-HexNAc-Hex-Hex sequence (Fig. 6, *B* and *C*). The ^0,2^A_5_ ion at *m/z* 1335 demonstrated 4-substitution of the innermost HexNAc. Thus, a branched decasaccharide with P1 terminals (Galα4Galβ4GlcNAcβ3(Galα4Galβ4G lcNAcβ6)Galβ4GlcNAcβ3Galβ4Glc) was tentatively identified.

**Figure 7:**
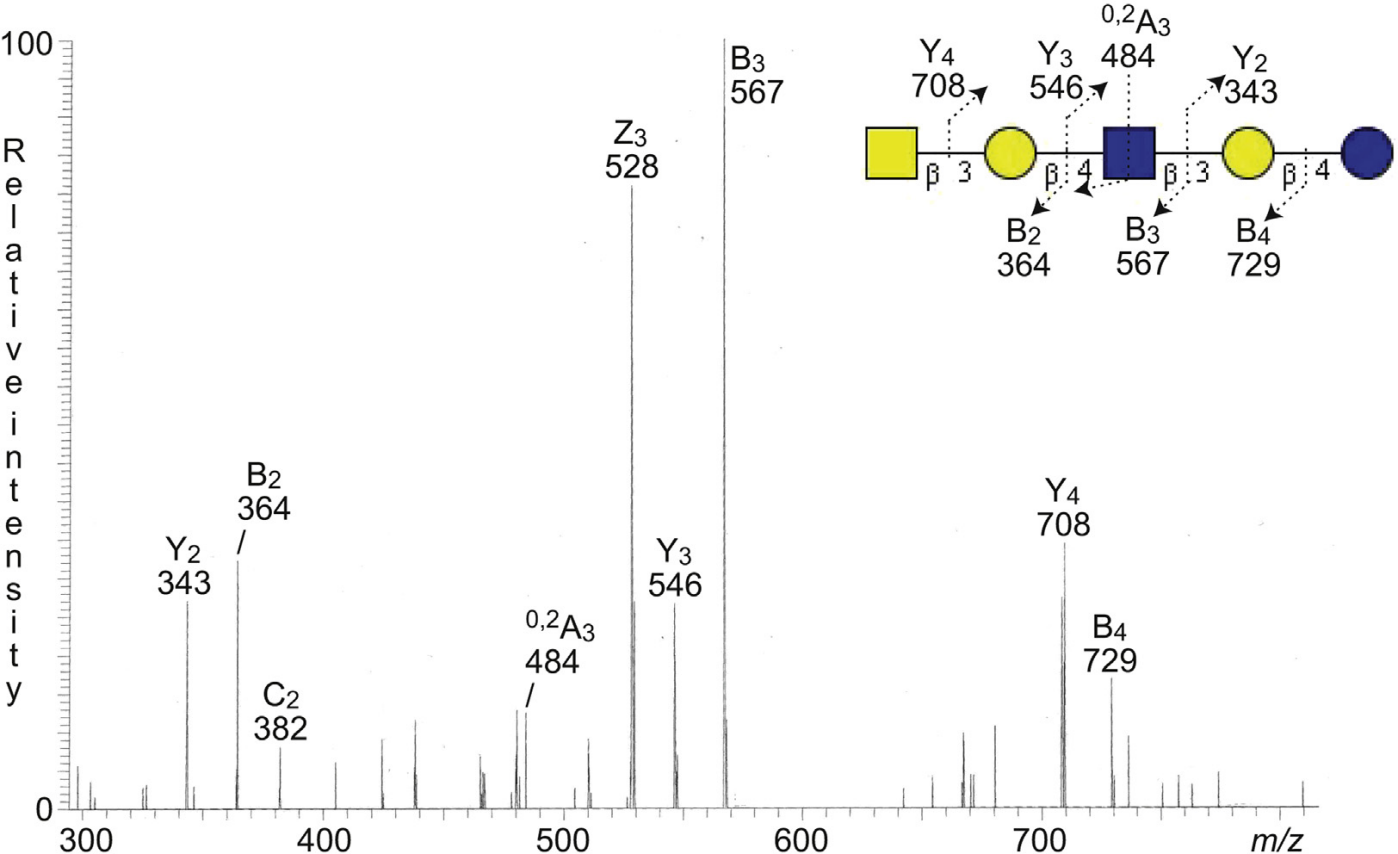
**LC–ESI/MS of the reduced oligosaccharides derived from fraction GC-3 by hydrolysis with endoglycoceramidase II from *Rhodococcus* spp.** MS^2^ of the ion at *m/z* 911 at retention time 15.5 min. The glycosphingolipid-derived oligosaccharides from fraction GC-3, obtained by endoglycoceramidase II hydrolysis, were reduced by treatment with sodium borohydride. The identification of oligosaccharide was based on their retention times, determined molecular masses, and subsequent MS^2^ sequencing. The proposed structure in the interpretation formula is depicted using the Symbol Nomenclature for Glycomics (SNFG) (56, 57), and nomenclature of fragments was defined by Domon and Costello (58). ESI, electrospray ionization; LC, liquid chromatography; MS, mass spectrometry.

The base peak chromatogram also had a minor molecular ion at *m/z* 909, eluting at 33.6 min (data not shown). Here, the MS^2^ spectrum was very weak and did not allow a reliable interpretation of the carbohydrate sequence. Therefore, the sample was reduced and reanalyzed by LC–ESI/MS. The spectrum obtained by MS^2^ of the ion at *m/z* 911 (reduced *m/z* 909) (Fig. 7) had a number of Y and Z ions (Y_2_ at *m/z* 343, Z_3_ at *m/z* 528, Y_3_ at *m/z* 546, and Y_4_ at *m/z* 708), which along with the series of B and C ions (B_2_ at *m/z* 364, C_2_ at *m/z* 382, B_3_ at *m/z* 567, and B_4_ at *m/z* 729), identified a HexNAc-Hex-HexNAc-Hex-Hex sequence. The ^0,2^A_3_ fragment ion at *m/z* 484 demonstrated that the internal HexNAc was substitued at C-4, that is, a type 2 chain. Taken together, this gave identification of an x_2_ pentasaccharide (GalNAcβ3Galβ4GlcNAcβ3Galβ4Glc).

### α-galactosidase treatment and LC–ESI/MS of fraction GC-3

**Figure 8:**
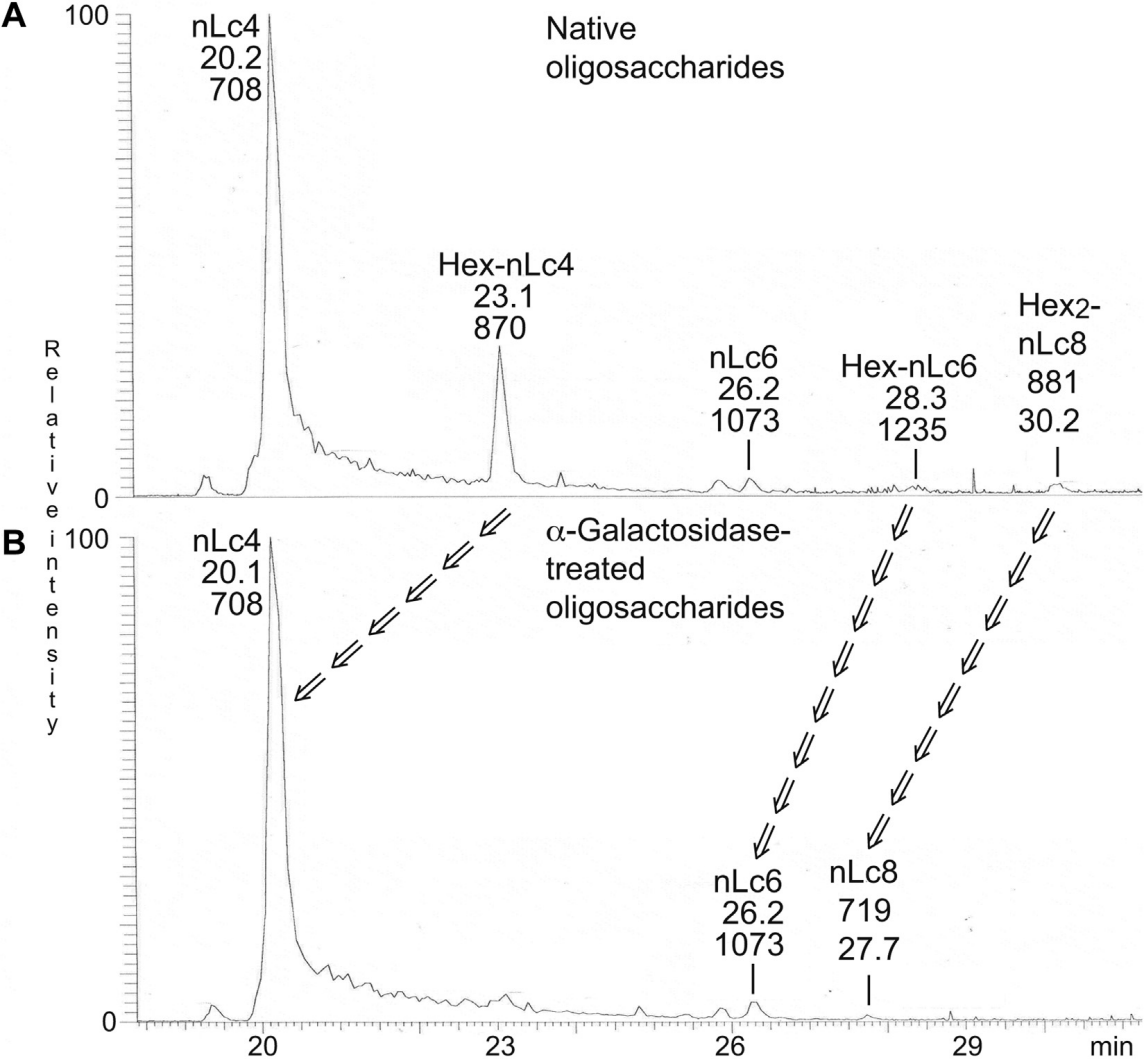
**α-galactosidase hydrolysis.** The glycosphingolipid-derived oligosaccharides from fraction GC-3, obtained by endoglycoceramidase II hydrolysis, were reduced by treatment with sodium borohydride, and part of the reduced samples was digested with green coffee bean α-galactosidase. The identification of oligosaccharide was based on their retention times, determined molecular masses, and subsequent MS^2^ sequencing. *A*, base peak chromatogram from LC–ESI/MS of the reduced oligosaccharides obtained by digestion of fraction GC-3 with *Rhodococcus* endoglycoceramidase II. *B*, base peak chromatogram from LC–ESI/MS after α-galactosidase hydrolysis of the reduced oligosaccharides obtained by digestion of fraction GC-3 with *Rhodococcus* endoglycoceramidase II. The *arrows* denote parent and related product glycans. Treatment with α-galactosidase gave removal of terminal Galα (162 Da) from *m/z* 870 (P1 pentasaccharide), *m/z* 1235 (P1 heptasaccharide), and *m/z* 881/1762 (P1 decasaccharide) in (*A*), resulting in molecular ions at *m/z* 708, *m/z* 1073, and *m/z* 719/1438 in (*B*). The oligosaccharides identified in the chromatograms were: nLc4, Galβ4GlcNAcβ3Galβ4Glc; Hex-nLc4, Galα4Galβ4GlcNAcβ3Galβ4Glc; nLc6, Galβ4GlcNAcβ3Galβ4GlcNAcβ3Galβ4Glc; Hex_2_-nLc8, Galα4Galβ4GlcNAcβ3(Galα4Galβ4GlcNAcβ6)Galβ4GlcNAcβ3Galβ4Glc; Hex-nLc6, Galα4Galβ4GlcNAcβ3Galβ4GlcNAcβ3Galβ4Glc; nLc8, Galβ4GlcNAcβ3(Galβ4GlcNAcβ6)Galβ4GlcNAcβ3Galβ4Glc. ESI, electrospray ionization; LC, liquid chromatography; MS, mass spectrometry.

Three oligosaccharides with terminal Hex-Hex-HexNAc sequence were identified in fraction GC-3 (*m/z* 868 Hex-Hex-HexNAc-Hex-Hex; *m/z* 1233 Hex-Hex-HexNAc-Hex-HexNAc-Hex-Hex; *m/z* 880/*m/z* 1760 Hex-Hex-HexNAc-(Hex-Hex-HexNAc)Hex-HexNAc-Hex-Hex). We speculated that these were the P1 pentasaccharide and a heptasaccharide and decasaccharide with P1 terminals. To substantiate this speculation, the oligosaccharides from the reduced fraction GC-3 were digested with green coffee bean α-galactosidase, which releases nonreducing terminal α(3,4,6)-linked galactose from oligosaccharides. The resulting oligosaccharides were analyzed by LC–ESI/MS (Fig. 8*B*) and compared with the untreated oligosaccharides (Fig. 8*A*) from fraction GC-3.

Upon treatment with α-galactosidase, the ions at *m/z* 870 (reduced *m/z* 868; Hex-Hex-HexNAc-Hex-Hex pentasaccharide), *m/z* 1235 (reduced *m/z* 1233; Hex-Hex-HexNAc-Hex-HexNAc-Hex-Hex heptasaccharide), and *m/z* 881/1762 (reduced *m/z* 880/1760; Hex-Hex-HexNAc-(Hex-Hex-HexNAc)Hex-HexNAc-Hex-Hex decasaccharide) disappeared (Fig. 8*B*). Since removal of terminal Galα (162 Da) from *m/z* 870 and *m/z* 1235 give *m/z* 708 and *m/z* 1073, respectively, no novel ions appeared in these cases. MS^2^ of the ion at *m/z* 706 at retention time 20.1 min identified a neolacto tetrasaccharide, and MS^2^ of the ion at *m/z* 1071 at retention time 26.2 min demonstrated a neolacto hexasaccharide (data not shown).

**Figure 9:**
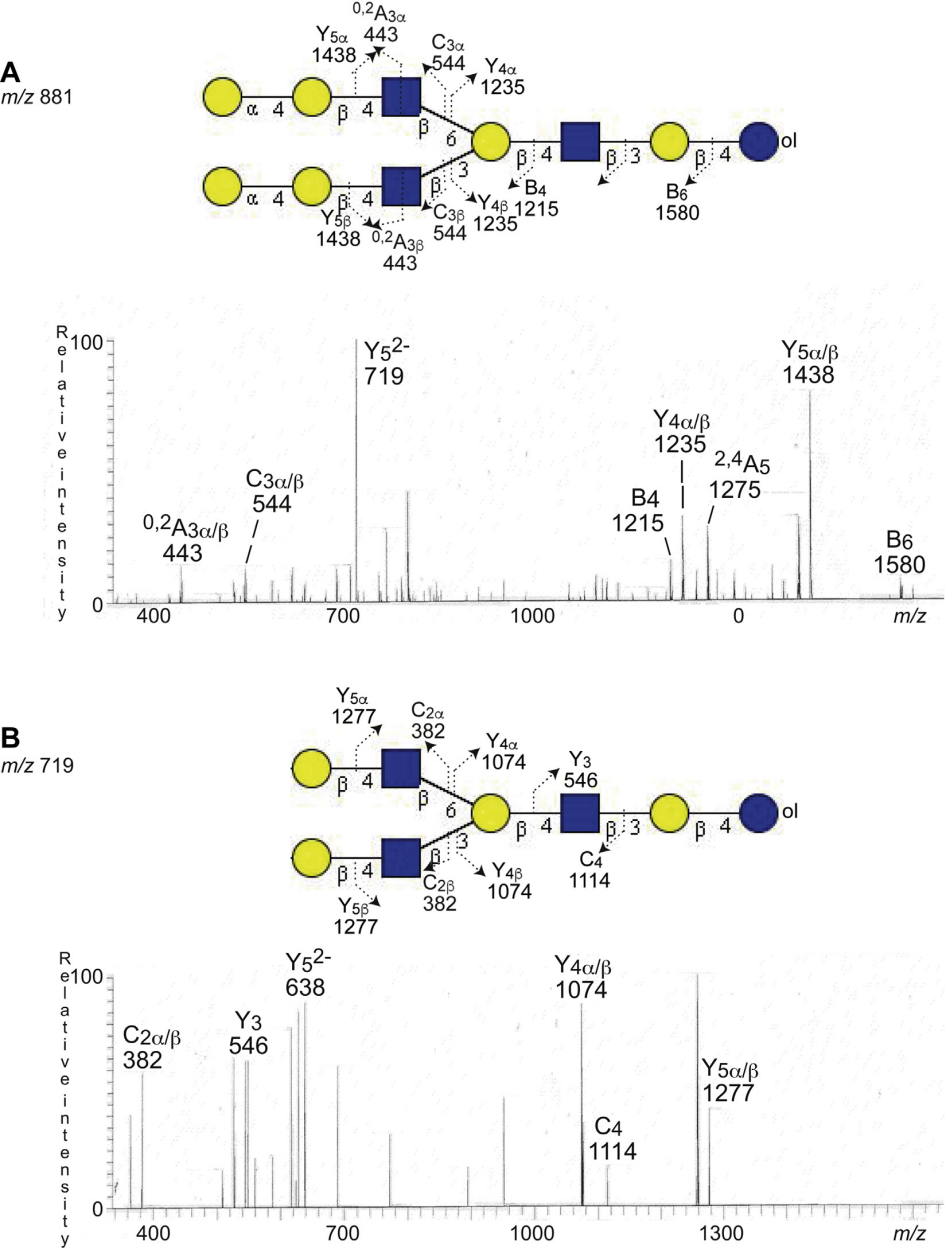
**α-galactosidase hydrolysis.** The glycosphingolipid-derived oligosaccharides from fraction GC-3, obtained by endoglycoceramidase II hydrolysis, were reduced by treatment with sodium borohydride, and part of the reduced samples was digested with green coffee bean α-galactosidase. The identification of oligosaccharide was based on their retention times, determined molecular masses, and subsequent MS^2^ sequencing. *A*, MS^2^ of the ion at *m/z* 881/1762 at retention time of 30.2 min from LC–ESI/MS of the reduced oligosaccharides obtained by digestion of fraction GC-3 with *Rhodococcus* endoglycoceramidase II. *B*, MS^2^ of the ion at *m/z* 718/1436 at retention time 27.7 min from LC–ESI/MS after α-galactosidase hydrolysis of the reduced oligosaccharides obtained by digestion of fraction GC-3 with *Rhodococcus* endoglycoceramidase II. The proposed structures in the interpretation formulas are depicted using the Symbol Nomenclature for Glycomics (SNFG) (56, 57), and nomenclature of fragments was defined by Domon and Costello (58). ESI, electrospray ionization; LC, liquid chromatography; MS, mass spectrometry.

In the α-galactosidase-treated sample, there was also a novel ion at *m/z* 719/1438 (Fig. 8*B*), corresponding to removal of two terminal Galα (162 Da x 2) from *m/z* 881/1762. MS^2^ of the ion at *m/z* 881/1762 in the untreated sample gave B-type and C-type fragment ions (C_3α/β_ at *m/z* 544, B_4_ at *m/z* 1215, and B_6_ at *m/z* 1580) and Y ions (Y_4α/β_ at *m/z* 1235 and Y_5α/β_ at *m/z* 1438) identifying a Hex-Hex-HexNAc-(Hex-Hex-HexNAc)Hex-HexNAc-Hex-Hex decasaccharide, as aforementioned (Fig. 9*A*). There was also a ^0,2^A_3α/β_ fragment ion at *m/z* 443 demonstrating C-4 substitution of the HexNAcs close to the nonreducing ends, that is, type 2 chains.

The MS^2^ spectrum of the novel ion at *m/z* 719/1438 (Fig. 9*B*) had a C_2α/β_ ion at *m/z* 382 demonstrating Hex-HexNAc terminals and a series of Y and Z ions (Y_3_ at *m/z* 546, Y_4α/β_ at *m/z* 1074, Z_5α/β_ at *m/z* 1258, and Y_5α/β_ at *m/z* 1277). Taken together, this demonstrated a Hex-HexNAc-(Hex-HexNAc)Hex-HexNAc-Hex-Hex octasaccharide.

Thus, the hydrolysis with α-galactosidase demonstrated that the terminal Hexs of the Hex-Hex-HexNAc-Hex-Hex pentasaccharide, Hex-Hex-HexNAc-Hex-HexNAc-Hex-Hex heptasaccharide, and Hex-Hex-HexNAc-(Hex-Hex-HexNAc)Hex-HexNAc-Hex-Hex decasaccharide were α-linked.

### Binding of antibodies and lectins to the nonacid subfractions from human gastric adenocarcinoma

**Figure 10:**
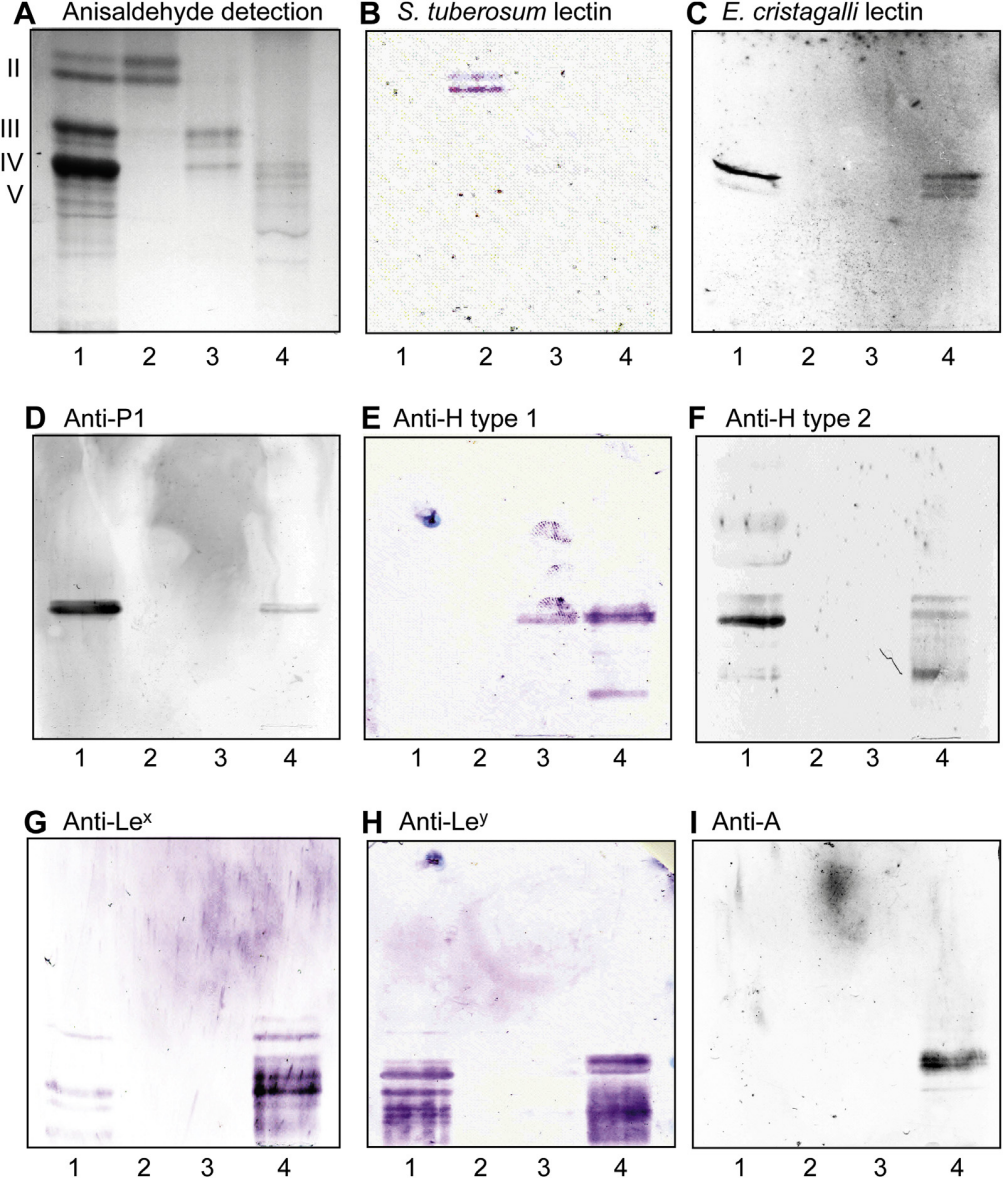
**Binding of antibodies and lectins to the subfractions of nonacid glycosphingolipids of human gastric cancer.** Thin layer chromatogram detected with anisaldehyde (*A*), and autoradiograms obtained by binding of *Solanum tuberosum* lectin (*B*), *Erythrina cristagalli* lectin (*C*), and monoclonal antibodies directed against blood group P1 (*D*), blood group H type 1 (*E*), blood group H type 2 (*F*), blood group Le^x^ (*G*), blood group Le^Y^ (*H*), and blood group A (*I*). The glycosphingolipids were separated on aluminum-backed silica gel plates, using chloroform/methanol/water 60:35:8 (by volume) as solvent system, and the binding assays were performed as described under the “Experimental procedures” section. The lanes were: lane 1, reference nonacid glycosphingolipids of human erythrocytes blood group O, 40 μg; lane 2, fraction GC-1, 4 μg; lane 3, fraction GC-2, 4 μg; and lane 4, fraction GC-3, 4 μg.

In order to validate the structural information obtained by MS, the binding of a number of carbohydrate-recognizing ligands to fractions GC-1 and GC-3 was thereafter examined in chromatogram binding assays (Fig. 10). The *Solanum tuberosum* lectin binds to lactosylceramide with sphingosine and nonhydroxy fatty acids (17). Thus, the binding of *S. tuberosum* lectin in the dihexosylregion in fraction GC-1 confirmed the presence of lactosylceramide with this ceramide composition (Fig. 10*B*, lane 2). The Galβ4GlcNAc/Fucα2Galβ4GlcNAc recognizing lectin from *Erythrina cristagalli* (18) gave three bands in fraction GC-3 (Fig. 10*C*, lane 4). The upper sharp band most likely was neolactotetraosylceramide, whereas the lower double band was the H type 2 pentaosylceramide (also shown in Fig. 10*F*).

The monoclonal antibodies directed against the P1 epitope bound in the pentaosylceramide region in fraction GC-3 (Fig. 10*D*). The presence of glycosphingolipids with blood group H type 1, H type 2, Le^x^, Le^y^, and A determinants in fraction GC-3 was confirmed by the binding of monoclonal antibodies (Fig. 10, *E*–*I*, lane 4). The antibodies against H type 1, H type 2, Le^x^, and Le^y^ also recognized slow-migrating compounds in fraction GC-3, indicating the presence of complex glycosphingolipids carrying these determinants.

Binding of *H. pylori* strain J99 and monoclonal antibodies directed against the Le^b^ determinant to fractions GC-1 and GC-3 was also tested, but no binding was obtained.

The nonacid glycosphingolipids identified in the human gastric adenocarcinoma are summarized in Table 2.

**Table 2:** Glycosphingolipids identified in the human gastric adenocarcinoma

*m/z*	Trivial name	Structure
503	Globotri (Gb3)	Galα4Galβ4Glcβ1Cer
544	Lactotri (Lc3)	GlcNAcβ3Galβ4Glcβ1Cer
706	Globotetra (Gb4)	GalNAcβ3Galα4Galβ4Glcβ1Cer
706	Lactotetra (Lc4)	Galβ3GlcNAcβ3Galβ4Glcβ1Cer
706	Neolactotetra (nLc4)	Galβ4GlcNAcβ3Galβ4Glcβ1Cerβ1Cer
852	H type 1 penta (H5-1)	Fucα2Galβ3GlcNAcβ3Galβ4Glcβ1Cer
852	H type 2 penta (H5-2)	Fucα2Galβ4GlcNAcβ3Galβ4Glcβ1Cer
852	Le^x^ penta (Le^x^-5)	Galβ4(Fucα3)GlcNAcβ3Galβ4Glcβ1Cer
868	P1 penta (P1)	Galα4Galβ4GlcNAcβ3Galβ4Glcβ1Cer
909	x_2_ penta (x_2_)	GalNAcβ3Galβ4GlcNAcβ3Galβ4Glcβ1Cer
998	Le^y^ hexa (Le^y^-6)	Fucα2Galβ4(Fucα3)GlcNAcβ3Galβ4Glcβ1Cer
1055	A hexa type 2 (A6-2)	GalNAcα3(Fucα2)Galβ4GlcNAcβ3Galβ4Glcβ1Cer
1071	Neolactohexa (nLc6)	Galβ4GlcNAcβ3Galβ4GlcNAcβ3Galβ4Glcβ1Cer
1233	P1 hepta	Galα4Galβ4GlcNAcβ3Galβ4GlcNAcβ3Galβ4Glcβ1Cer
880/1760	P1 deca	Galα4Galβ4GlcNAcβ3(Galα4Galβ4GlcNAcβ6)Galβ4GlcNAcβ3Galβ4Glcβ1Cer

## Discussion

There are three identified carbohydrate-binding *H. pylori* adhesins, the Le^b^-binding BabA adhesin, the sialic acid–binding SabA adhesin, and LabA with undefined carbohydrate-binding specificity. In addition, *H. pylori* HopQ protein functions as a carbohydrate-independent adhesin and binds to the N-terminal of human carcinoembryonic antigen–related cell adhesion molecules, which leads to translocation of the CagA pathogenicity island into host cells (19, 20).

In this study, the nonacid glycosphingolipids of a human gastric adenocarcinoma were characterized. The majority of the complex nonacid glycosphingolipids had type 2 (Galβ4GlcNAc) core chains, that is, the neolactotetraosylceramide, the Le^x^, H type 2, x_2_, and the P1 pentaosylceramides, and the Le^y^, A type 2, and neolacto hexaosylceramides. A predominance of glycosphingolipids with type 2 core was also present in the normal human stomachs (13). The gastric adenocarcinoma also had two compounds with type 1 (Galβ3GlcNAc) core; lactotetraosylceramide and the H type 1 pentaosylceramide. In addition, there were compounds with globo (GalαGal) core chains, that is, globotriaosylceramide and globotetraosylceramide. The glycan parts of the dihexosylceramides were not resolved in this study. However, in the healthy human stomach, the dihexosylceramides are a mixture of lactosylceramide and digalactosylceramide (13, 21).

There was no binding of *H. pylori* to the total nonacid glycosphingolipid fraction from the gastric adenocarcinoma, and this fraction had mainly neolactotetraosylceramide and the P1 glycosphingolipid. Minor potential *H. pylori* binding targets identified were lactotetraosylceramide (22), the H type 1 pentaosylceramide (23), the x_2_ pentaosylceramide, and neolactohexaosylceramide (24). Separation into subfractions was required for characterization of the minor complex fucosylated glycosphingolipids (Le^x^ and H type 2 pentaosylceramides as well as the Le^y^ and A type 2 hexaosylceramides). This is in contrast to the normal human stomach, where these compounds were characterized using the total nonacid glycosphingolipid fractions (13). Previous immunohistochemistry studies have demonstrated changes in expression of Lewis antigens upon malignant transformation in the stomach, with decreased levels of Le^b^ and increased levels of Le^a^ (25, 26, 27). A decrease in blood group ABO antigen expression in gastric cancer has also been reported (28).

Thus, the repertoire of nonacid glycosphingolipids in the gastric adenocarcinoma had both similarities and differences with the glycosphingolipids previously characterized in the normal human stomach (13), as summarized in Table 3. Several *H. pylori*-binding compounds (lactotetraosylceramide, neolactotetraosylceramide, the x_2_ and H type 1 pentaosylceramide, and neolactohexaosylceramide) were present in both cases. However, two main targets for BabA-mediated binding of *H. pylori*, the Le^b^ hexaosylceramide and blood group A type 1/ALe^b^ heptaosylceramide, were not found in the gastric adenocarcinoma, that is, the adhesion targets for BabA-mediated binding of *H. pylori* are reduced in gastric cancer. Together, our results support that the BabA-mediated adherence of *H. pylori* is primarily important for the initial adhesion of the bacteria to the healthy human stomach, which is crucial for colonization and subsequent infection.

**Table 3:** Comparison of glycosphingolipids in normal human stomach and human gastric adenocarcinoma

Trivial name	Glycosphingolipid structure	Normal stomach^a^	Gastric cancer	BabA binding^b^
Globotetra	GalNAcβ3Galα4Galβ4Glcβ1Cer	+	+	
Lactotetra	Galβ3GlcNAcβ3Galβ4Glcβ1Cer^c^	+	+	
Neolactotetra	Galβ4GlcNAcβ3Galβ4Glcβ1Cer^c^	+	+	
x_2_ penta	GalNAcβ3Galβ4GlcNAcβ3Galβ4Glcβ1Cer^c^	+	+	
H type 1 penta	Fucα2Galβ3GlcNAcβ3Galβ4Glcβ1Cer^c^	+	+	+
H type 2 penta	Fucα2Galβ4GlcNAcβ3Galβ4Glcβ1Cer	+	+	
Le^a^ penta	Galβ3(Fucα4)GlcNAcβ3Galβ4Glcβ1Cer	+	−	
Le^x^ penta	Galβ4(Fucα3)GlcNAcβ3Galβ4Glcβ1Cer	+	+	
P1 penta	Galα4Galβ4GlcNAcβ3Galβ4Glcβ1Cer^d^	−	+	
Neolactohexa	Galβ4GlcNAcβ3Galβ4GlcNAcβ3Galβ4Glcβ1Cer^c^	+	+	
Le^b^ hexa	Fucα2Galβ3(Fucα4)GlcNAcβ3Galβ4Glcβ1Cer^c^	+	−	+
Le^y^ hexa	Fucα2Galβ4(Fucα3)GlcNAcβ3Galβ4Glcβ1Cer	+	+	
A type 2 hexa	GalNAcα3(Fucα2)Galβ4GlcNAcβ3Galβ4Glcβ1Cer	+	+	
H type 2 hepta	Fucα2Galβ4GlcNAcβ3Galβ4GlcNAcβ3Galβ4Glcβ1Cer	+	−	
A type 1 hepta	GalNAcα3(Fucα2)Galβ3(Fucα4)GlcNAcβ3Galβ4Glcβ1Cer^c^	+	−	+
A type 2 hepta	GalNAcα3(Fucα2)Galβ4(Fucα3)GlcNAcβ3Galβ4Glcβ1Cer	+	−	
A type 2 octa	GalNAcα3(Fucα2)Galβ4GlcNAcβ3Galβ4GlcNAcβ3Galβ4Glcβ1Cer	+	−	
P1 hepta	Galα4Galβ4GlcNAcββ3Galβ4GlcNAcβ3Galβ4Glcβ1Cer^d^	−	+	
P1 deca	Galα4Galβ4GlcNAcβ3(Gala4Galβ4GlcNAcβ6)Galβ4GlcNAcβ3Galβ4Glcβ1Cer^d^	−	+	

a Data from the blood group A(Rh+)P human stomach reported in Ref. (14).

b Compounds recognized by *H. pylori* BabA adhesin.

c The compounds have been characterized as *H. pylori* binding in prevoius studies (reviewed in Ref. ((3).

d Marks the compounds present in the human gastric adenocarcinoma only.

There are several reports of human gastric adenocarcinoma glycosphingolipids from the 1970 to 1980ies (29, 30, 31, 32, 33). These studies were focused on incompatible blood group antigens, that is, blood group A and/or and Forssman glycosphingolipids in tumors from blood group O and B individuals. In many cases, polyclonal antibodies were used, and thus to some extent, these findings may be due to crossreactivities with the Tn antigen, since the blood group A antigen, the Forssman determinant, and the Tn antigen all have a terminal α3-linked GalNAc residue (34, 35). However, in some cases, solid chemical evidence demonstrated the presence of blood group A glycosphingolipids in tumors from blood group O individuals (30, 32). The role of such incompatible blood group A antigens in the tumorigenic process is currently not known.

Changes in glycosylation is one hallmark of cancer and are due to abnormally expressed glycosyltransferases and glycosidases in tumor cells, leading to the generation of tumor-associated carbohydrate antigens (36, 37, 38, 39). In gastric tumors, the occurrence of truncated O-glycans has been reported (40, 41), and this is associated with cancer aggressiveness and poor prognosis (41, 42). An enhanced expression of sialylated Le^a^ has also been found in gastric cancers (25).

In this study, the identification of glycosphingolipids with the P1 terminal among the gastric adenocarcinoma glycosphingolipids was an unexpected finding, since the P1 pentaosylceramide is mainly expressed on human erythrocytes (43), and was not identified in the normal human stomachs (13). The P1 glycosphingolipid has, however, been identified as a marker of ovarian cancer (44).

The characterization of the P1 pentaosylceramide, and the heptaosylceramide and branched decaosylceramide with P1 terminals, was based on:

(i) Identification of terminal Hex-Hex-HexNAc sequences by LC–ESI/MS.
(ii) Binding of monoclonal antibodies directed against the P1 epitope in the pentaosylceramide region.
(iii) A ^0,2^A_2_ fragment ion at *m/z* 281 obtained by MS^3^, which demonstrated that the subterminal Hex was substituted at C-4 (only seen for the pentaosylceramide).
(iv) Disappearance of the molecular ions corresponding to the pentaosylceramide, heptaosylceramide, and decaosylceramide upon treatment with α-galactosidase.

Furthermore, terminal α3-linked Gal is not likely since no linear Galα3-terminated glycoconjugates are present in human tissues. This is due to point mutations in the human gene for the α1,3galactosyltransferase, which results in a frameshift and a premature stop codon (45).

The heptaosylceramide and branched decaosylceramide with P1 terminals are to our knowledge novel glycosphingolipid structures and the first characterization of complex glycosphingolipids larger than pentaosylceramide carrying the P1 trisaccharide.

*H. pylori* binds to several glycosphingolipids with neolacto core chain, as for example, the B5 pentaosylceramide and the x_2_ pentaosylceramide (24). However, the P1 glycosphingolipid is not recognized by *H. pylori* (24), and thus, the P1 glycosphingolipids in gastric cancer are not novel adhesion targets for the bacteria.

Interestingly, in 1976, Levine (46) reported about a gastric adenocarcinoma in a woman with the rare genotype *pp*, lacking the P1 antigen. Prior to surgery, this patient was given a transfusion with incompatible blood, and thereby, her titers of anti-P1 antibodies increased from 1:4 to 1:512. The 66-year-old patient survived for 22 years and died from natural causes with no evidence of metastases. Subsequent analysis of the glycosphingolipids in the tumor material demonstrated the presence of a compound, which comigrated with the P1 pentaosylceramide on thin layer chromatograms, and was degraded by α-galactosidase (47). Thus, our finding of the P1 glycosphingolipids in the gastric adenocarcinoma, along with this classical anecdotal report, suggests that further studies should be done to investigate the potential role of P1 as a diagnostic and prognostic biomarker for gastric cancer, and target for anticancer immunotherapeutics.

## Experimental procedures

### Glycosphingolipid preparations

The study was conducted according to the tenets of the Declaration of Helsinki. The gastric adenocarcinoma was collected in the 1970ies at Sahlgrenska University Hospital, Göteborg, Sweden (before the hospital had an ethics committee). The tissue (dry weight 80 g) was obtained at autopsy, and after lyophilization, the tissue was kept at −70 °C for several years. The isolation of total acid and total nonacid glycosphingolipids has been described (14). In brief, the lyophilized tissue was extracted in a Soxleth apparatus with mixtures of chloroform and methanol (2:1 and 1:9, by volume). The resulting material was pooled and subjected to mild alkaline hydrolysis followed by dialysis. Thereafter, nonpolar compounds were removed by chromatography on a silicic acid column. Acid and nonacid glycosphingolipids were separated by ion change chromatography on a diethylaminoethyl-cellulose column. In order to separate the nonacid glycosphingolipids from alkali-stable phospholipids, the nonacid fractions were then acetylated and separated on a second silicic acid column, followed by deacetylation and dialysis. Final purifications are performed by chromatography on diethylaminoethyl-cellulose and silicic acid columns.

After the first characterization by binding assays and LC–ESI/MS, the nonacid glycosphingolipids were separated on an Iatrobeads column eluted with increasing volumes of methanol in chloroform. The fractions obtained were analyzed by thin layer chromatography and anisaldehyde and thereafter pooled according to their mobility on thin layer chromatograms, resulting in three subfractions, which were denoted fractions GC-1, GC-2, and GC-3.

### Reference glycosphingolipids

Total acid and nonacid glycosphingolipid fractions were isolated as described (48). Individual glycosphingolipids were isolated by repeated chromatography on silicic acid columns and by HPLC and identified by MS (15, 49) and ^1^H-NMR spectroscopy (50).

### Thin-layer chromatography

Thin-layer chromatography was performed on aluminium- or glass-backed silica gel 60 high-performance thin-layer plates (Merck). Glycosphingolipid mixtures (40 μg), or pure glycosphingolipids (4 μg), were applied to the plates and chromatographed using chloroform/methanol/water 60:35:8 (by volume) as solvent system. Chemical detection was done with anisaldehyde (51).

### Chromatogram binding assays

The carbohydrate-binding ligands and dilutions used in the chromatogram binding assays are given in Table 4. Binding of antibodies to glycosphingolipids separated on thin-layer chromatograms was performed as described by Barone *et al.* (52). After elution, the dried thin-layer plates were treated with a mixture of 0.5% polyisobutylmethacrylate (w/v) in diethylether/*n*-hexane (5:1, v/v) for 1 min and then air-dried. Thereafter, followed by a 2 h incubation at room temperature with PBS (pH 7.3) containing 2% (w/v) bovine serum albumin, 0.1% (w/v) NaN_3_, and 0.1% (w/v) Tween-20 (solution A) to reduce unspecific binding. Then, the chromatograms were incubated for 2 h at room temperature with suspensions of monoclonal antibodies diluted in solution A, followed by washings with PBS.

**Table 4:** Carbohydrate-binding ligands used in chromatogram binding assays

Ligand	Clone/designation	Manufacturer/reference	Specificity	Dilution
Anti-P1	P3NIL100	Immucor Gamma	Galα4Galβ4GlcNAc	1:100
Anti-A	HE-195	Sigma–Aldrich	GalNAcα3(Fucα2)Gal	1:500
Anti-H type 1	17-206	GeneTex/Abcam	Fucα2Galβ3GlcNAc	1:100
Anti-H type 2	A583	Dakopatts	Fucα2Galβ4GlcNAc	1:100
Anti-Lewis^x^	P12	Santa Cruz Biotechnology	Galβ4(Fucα3)GlcNAc	1:200
Anti-Lewis^y^	F3	GeneTex/Abcam	Fucα2Galβ4(Fucα3)GlcNAc	1:100
Anti-Lewis^b^	T218	Santa Cruz Biotechnology	Fucα2Galβ3(Fucα4)GlcNAc	1:100
P-fimbriated *Escherichia coli*	—	Ref. (21)	Galα4Gal	—
*Helicobacter pylori* strain J99	—	Ref. (6)	Fucα2Galβ3(Fucα4)GlcNAc Galβ3GlcNAc	—
*Helicobacter pylori* strain P12	—	Benktander *et al.*, in article	Galβ3GlcNAc	—
*Erytrina christagalli* lectin	—	Vector Laboratories, Inc	Galβ4GlcNAc Fucα2Galβ4GlcNAc	1:100
*Solanum tuberosum* lectin	—	bioWORLD	Galβ4Glc/Galβ4GlcNAc	1:100

Two types of secondary antibodies were used for detection. The first type was ^125^I-labeled (labeled by the Iodogen method according to the manufacturer's [Pierce; catalog no.: 28600] instructions) rabbit antimouse antibodies diluted to 2 × 10^6^ cpm/ml in solution A, which were incubated for 2 h. Thereafter, the plates were washed six times with PBS. Dried chromatograms were then autoradiographed for 12 to 24 h using XAR-5 X-ray films (Carestream; catalog no.: 8941114).

The other type of secondary antibodies used was alkaline phosphate–conjugated goat antimouse antibodies (Sigma–Aldrich; catalog no.: A0162) at a dilution of 1: 500 in solution A, which were incubated for 1 h. Alkaline phosphate–conjugated goat antihuman immunoglobulin M antibodies (Sigma–Aldrich; catalog no.: A3437), at a dilution of 1:400 in solution A, were used for detection of anti-P1 antibodies. The reactions were visualized with 5-bromo-4-chloro-3-indolyl phosphate/nitro blue tetrazolium chromogenic substrate (Sigma–Aldrich; catalog no.: B5655-25TAB).

Binding of ^35^S-labeled *H. pylori* and P-fimbriated *E. coli* to glycosphingolipids on thin layer chromatograms was done as described (13, 21).

Binding of ^125^I-labeled *E. cristagalli* lectin (Sigma–Aldrich) to glycosphingolipids on thin layer chromatograms was done as described (18). Chromatogram binding assays with alkaline phosphate–conjugated *S. tuberosum* lectin (bioWORLD) were done as described (17), and 5-bromo-4-chloro-3-indolyl phosphate/nitro blue tetrazolium chromogenic substrate was used for visualization.

### LC–ESI/MS of native glycosphingolipids

The native glycosphingolipid fractions were analyzed by LC–ESI/MS as described (53). Aliquots of the glycosphingolipid fractions were dissolved in methanol:acetonitrile in proportion 75:25 (by volume) and separated on a 200 × 0.250 mm column, packed in-house with 5 μm polyamine II particles (YMC Europe GmbH). An autosampler, HTC-PAL (CTC Analytics AG), equipped with a cheminert valve (0.25 mm bore) and a 2 μl loop, was used for sample injection. An Agilent 1100 binary pump (Agilent Technologies) delivered a flow of 250 μl/min, which was split down in an 1/16” microvolume-T (0.15 mm bore) (Vici AG International) by a 50 cm × 50 μm i.d. fused silica capillary before the injector of the autosampler, allowing approximately 2 to 3 μl/min through the column. Samples were eluted with an aqueous gradient (A: 100% acetonitrile to B: 10 mM ammonium bicarbonate). The gradient (0–50% B) was eluted for 40 min, followed by a wash step with 100% B, and equilibration of the column for 20 min. The samples were analyzed in negative ion mode on a linear trap quadrupole (LTQ) ion mass spectrometer (Thermo Electron), with an IonMax standard ESI source equipped with a stainless steel needle kept at −3.5 kV. Compressed air was used as nebulizer gas. The heated capillary was kept at 270 °C, and the capillary voltage was −50 kV. Full scan (*m/z* 600–1800, two microscans, maximum 100 ms, and target value of 30,000) was performed, followed by data-dependent MS^2^ scans (two microscans, maximum of 100 ms, and target value of 10,000) with normalized collision energy of 35%, isolation window of 2.5 units, activation q = 0.25, and activation time of 30 ms). The threshold for MS^2^ was set to 500 counts.

Data acquisition and processing were conducted with Xcalibur software (Thermo Scientific; version 2.0.7). Manual assignment of glycosphingolipid sequences was done with the assistance of the Glycoworkbench tool (version 2.1) (54), and by comparison of retention times and MS^2^ spectra of reference glycosphingolipids.

### Endoglycoceramidase digestion and LC–ESI/MS

Endoglycoceramidase II from *Rhodococcus* spp. (Takara Bio Europe S.A.) was used for hydrolysis of the nonacid glycosphingolipids. The glycosphingolipids (50 μg) were resuspended in 100 μl 0.05 M sodium acetate buffer, pH 5.0, containing 120 μg sodium cholate, and sonicated briefly. Thereafter, 1 mU of enzyme was added, and the mixture was incubated at 37 °C for 48 h. The reaction was stopped by addition of chloroform/methanol/water to the final proportions 8:4:3 (by volume). The oligosaccharide-containing upper phase thus obtained was separated from detergent on a Sep-Pak QMA cartridge (Waters). The eluant containing the oligosaccharides was dried under nitrogen and under vacuum.

Part of the oligosaccharide samples was reduced by adding 20 μl of 200 mM NaBH_4_ in 50 mM KOH to the samples and incubating at 50 °C for 2 h (15). The samples were then acidified by adding 1 μl of glacial acetic acid, and the oligosaccharides were desalted by cation exchange chromatography and thereafter evaporated to dryness.

To characterize anomeric configuration of the terminal Hex-Hex sequence, part of the reduced oligosaccharide samples was digested with α-galactosidase (8 U) from green coffee bean (New England Biolabs), which releases nonreducing terminal α(3,4,6)-linked galactose from oligosaccharides, following the protocol of the manufacturer. Thereafter, the oligosaccharides were desalted using graphitized carbon solid-phase extraction as described (55).

The glycosphingolipid-derived oligosaccharides were resuspended in 50 μl water and analyzed by LC–ESI/MS as described (15). The oligosaccharides were separated on a column (100 × 0.250 mm) packed in-house with 5 μm porous graphite particles (Hypercarb, Thermo-Hypersil). An autosampler, HTC-PAL (CTC Analytics AG) equipped with a cheminert valve (0.25 mm bore) and a 2 μl loop, was used for sample injection. An Agilent 1100 binary pump (Agilent Technologies) delivered a flow of 250 μl/min, which was split down in an 1/16” microvolume-T (0.15 mm bore) (Vici AG International) by a 50 cm × 50 μm i.d. fused silica capillary before the injector of the autosampler, allowing approximately 3 to 5 μl/min through the column. The oligosaccharides (3 μl) were injected on to the column and eluted with an acetonitrile gradient (A: 10 mM ammonium bicarbonate; B: 10 mM ammonium bicarbonate in 80% acetonitrile). The gradient (0–45% B) was eluted for 46 min, followed by a wash step with 100% B, and equilibration of the column for 24 min. A 30 cm × 50 μm i.d. fused silica capillary was used as transfer line to the ion source.

The oligosaccharides were analyzed in negative ion mode on an LTQ ion mass spectrometer. The IonMax standard ESI source on the LTQ mass spectrometer was equipped with a stainless steel needle kept at −3.5 kV. Compressed air was used as nebulizer gas. The heated capillary was kept at 270 °C, and the capillary voltage was −50 kV. Full scan (*m/z* 380–2000, two microscans, maximum 100 ms, and target value of 30,000) was performed, followed by data-dependent MS^2^ scans of the three most abundant ions in each scan (2 microscans, maximum 100 ms, and target value of 10,000). The threshold for MS^2^ was set to 500 counts. Normalized collision energy was 35%, and an isolation window of 3 u, an activation q = 0.25, and an activation time of 30 ms, were used. Data acquisition and processing were conducted with Xcalibur software (Thermo Scientific; version 2.0.7).

Manual assignment of glycan sequences was done on the basis of knowledge of mammalian biosynthetic pathways, with the assistance of the Glycoworkbench tool (version 2.1) (54), and by comparison of retention times and MS^2^ spectra of oligosaccharides from reference glycosphingolipids (15).

## Data availability

Raw data were uploaded on Glycopost (https://glycopost.glycosmos.org/entry/GPST000232), accessed on December 17, 2021.

## Supporting information

This article contains supporting information.

## Conflict of interest

The authors declare that they have no conflicts of interest with the contents of this article.
