# Supplementary material for: Characterization of novel nonacid glycosphingolipids as biomarkers of human gastric adenocarcinoma
Source: J Biol Chem. 2022 Feb 15;298(4):101732. doi: 10.1016/j.jbc.2022.101732 (PMC8933711; doi:10.1016/j.jbc.2022.101732)
Supplement: Supplemental Figure S1 [file mmc1.pdf]

SUPPORTING INFORMATION

Non-acid glycosphingolipids of human gastric adenocarcinoma

Chunsheng Jin and Susann Teneberg

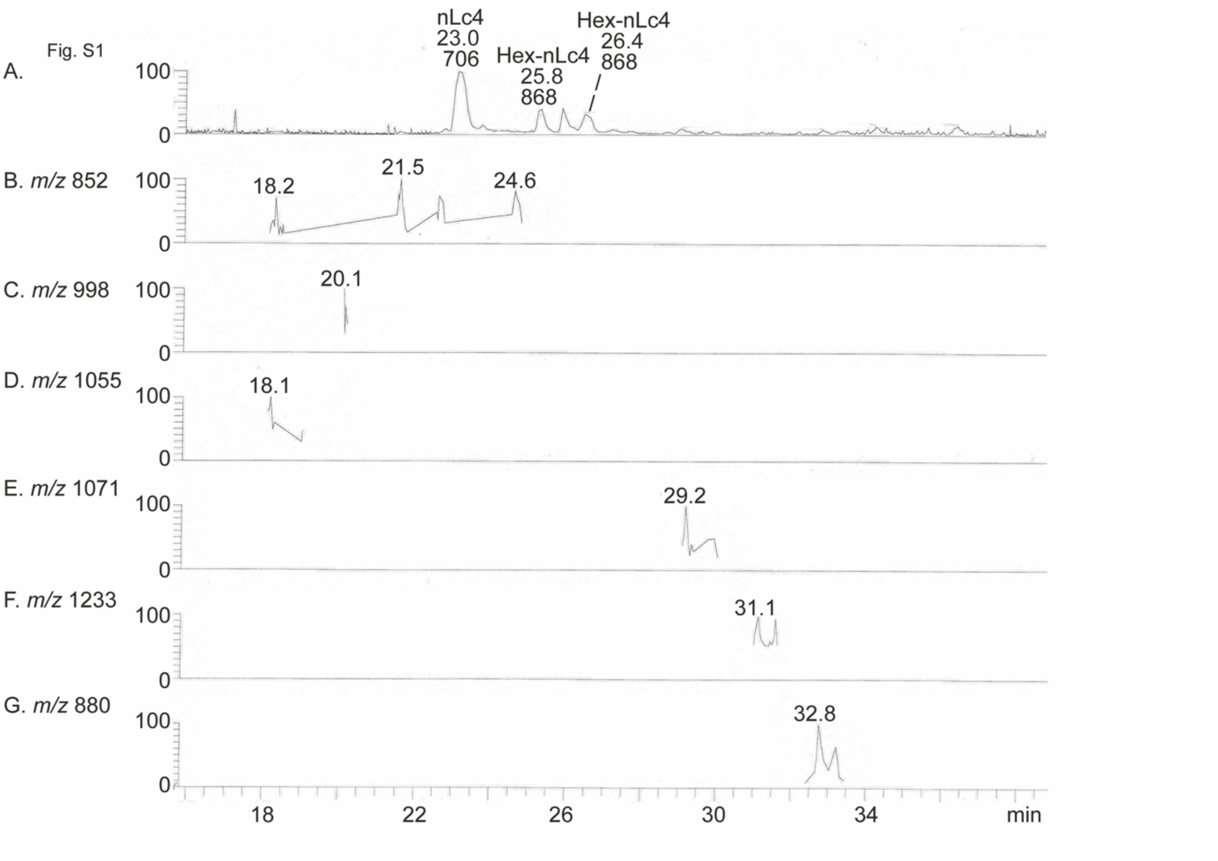

Supplementary Fig. S1. LC-ESI/MS of the oligosaccharides derived from fraction GC-3 from human gastric adenocarcinoma by hydrolysis with endoglycoceramidase II from *Rhodococcus* spp.

(A) Base peak chromatogram from LC-ESI/MS the oligosaccharides obtained from fraction GC-3.

(B) Reconstructed ion chromatogram of *m/z* 852.

(C) Reconstructed ion chromatogram of *m/z* 998.

(D) Reconstructed ion chromatogram of *m/z* 1055.

(E) Reconstructed ion chromatogram of *m/z* 1071.

(F) Reconstructed ion chromatogram of *m/z* 1233.

(G) Reconstructed ion chromatogram of *m/z* 880.

The oligosaccharides identified in the chromatogram were: nLc4, Galβ4GlcNAcβ3Galβ4Glc; Hex-nLc4; Hex-Galβ4GlcNAcβ3Galβ4Glc. Y-axis; Relative intensity.
